# Resveratrol Modulates the Redox Response and Bile Acid Metabolism to Maintain the Cholesterol Homeostasis in Fish *Megalobrama amblycephala* Offered a High-Carbohydrate Diet

**DOI:** 10.3390/antiox12010121

**Published:** 2023-01-03

**Authors:** Yaping Ge, Ling Zhang, Weiliang Chen, Miao Sun, Wenbin Liu, Xiangfei Li

**Affiliations:** Key Laboratory of Aquatic Nutrition and Feed Science of Jiangsu Province, College of Animal Science and Technology, Nanjing Agricultural University, No. 1 Weigang Road, Nanjing 210095, China

**Keywords:** redox balance, cholesterol homeostasis, bile acid metabolism, carbohydrate utilization, resveratrol, fish culture

## Abstract

This study aimed to characterize the effects of resveratrol on the redox balance, cholesterol homeostasis and bile acid metabolism of *Megalobrama amblycephala* offered a high-carbohydrate diet. Fish (35.0 ± 0.15 g) were fed four diets including one control diet (32% nitrogen-free extract), one high-carbohydrate diet (45% nitrogen-free extract, HC), and the HC diet supplemented with different levels (0.04%, HCR1; 0.08%, HCR2) of resveratrol for 12 weeks. The HC diet-induced redox imbalance is characterized by increased MDA content and decreased T-SOD and CAT activities in the liver. Resveratrol attenuated this by up-regulating the transcription of *Cu/Zn-sod*, and increasing the activities of T-SOD, CAT, and GPX. The HC diet enhanced the cholesterol synthesis, but decreased the bile acid synthesis via up-regulating both *hmgcr* and *acat2*, and down-regulating *cyp7a1*, thus resulting in excessive cholesterol accumulation. Resveratrol supplement decreased cholesterol synthesis, and increased cholesterol uptake in the liver by down-regulating both *hmgcr* and *acat2*, and up-regulating *ldlr*. It also increased bile acid synthesis and biliary excretion by up-regulating *cyp7a1*, and down-regulating *mrp2*, *oatp1*, and *oatp4* in the hindgut, thereby decreasing cholesterol accumulation. In conclusion, resveratrol improves the cholesterol homeostasis of *Megalobrama amblycephala* fed a high-carbohydrate diet by modulating the redox response and bile acid metabolism.

## 1. Introduction

The scale of aquaculture increases parallelly with the increase in fish consumption in recent years [1]. Meanwhile, a large amount of carbohydrates is frequently incorporated in aquafeeds to save the relatively expensive protein sources. However, the poor utilization of carbohydrates by fish makes them prone to an excessive accumulation of glycogen, cholesterol, and other lipids [2,3,4]. This inevitably leads to fatty liver and hepatobiliary syndromes in fish [5,6], as is a great challenge in the modern aquaculture industry. This not only has a negative impact on fish health but also further affects human health due to an excessive intake of dietary cholesterol [7]. Generally, compromised cholesterol homeostasis and dysfunctional bile acids metabolism are closely interrelated with the aforementioned syndromes in mammals [8,9]. However, the relevant information is still barely available in aquatic species.

Cholesterol, an ancient and fascinating molecule, plays an essential role in forming eukaryotic membranes. It accounts for approximately 30–40% of the total cellular lipids [10], thereby maintaining the rigidity, fluidity, and permeability of the membrane [11], as well as modulating membrane transport, signal transduction, and other cellular functions [12]. Cholesterol is mainly obtained from endogenous synthesis and exogenous uptake. Generally, the sterol regulatory element-binding protein 2 (SREBP2) is the master transcriptional regulator in cholesterol biosynthesis, which mainly happens in the liver. This process is assisted by more than 20 enzymes, among which both squalene monooxygenase and 3-hydroxy-3-methyl-glutaryl coenzyme A reductase (HMGCR) are rate-limiting ones [11]. After synthesis and intake, cholesterol is transported into the bloodstream as very low-density lipoproteins (VLDLs). The VLDLs are then converted into low-density lipoproteins (LDLs) [13], which are later uptaken by the peripheral cells through the low-density lipoproteins receptor (LDLR) in circulation. The remanent cholesterol is either esterified by the acyl coenzyme A: cholesterol acyltransferase (ACAT) into cholesterol esters or forms the high-density lipoproteins (HDLs) [14,15].

It is now widely acknowledged that cholesterol is also a precursor of bile acids. Generally, cholesterol is converted into primary bile acids with the assistance of both cholesterol 7α-hydroxylase (CYP7A1) and sterol 12α-hydroxylase (CYP8B1) in the liver [16]. Some of the primary bile acids bind with taurine or glycine to generate the conjugated bile acids under the action of various enzymes [17]. Then, facilitated by the multidrug resistance-associated protein 2 (MRP2) and the bile salt export pump (BSEP), the bile acids are secreted into the gallbladder and small intestine, respectively [18,19]. In the intestine, primary bile acids are converted into secondary bile acids by the intestinal flora [20]. Then more than 95% of the bile acids are returned to the liver via veins after reabsorption in the ileum [21], as is facilitated by the organic anion-transporting polypeptides (OATPs) and the Na^+^-taurocholate cotransporter (NTCP) in the hepatocellular sinusoids [22,23]. The aforementioned metabolic processes are mainly governed by the farnesoid X receptor α (FXRα), which is a nuclear receptor targeting various proteins like CYP8B1, CYP7A1, MRP2, OATPs, and so on to maintain the normal metabolism of bile acids [24]. However, this information is mainly characterized in mammals. The underlying mechanisms of bile acid metabolism in fish are still poorly interpreted.

Resveratrol (3,4′,5-trihydroxy-trans-stilbene, RSV) is a promising natural polyphenolic substance that can be used as a dietary supplement. As a plant antitoxin with abundant sources [25], resveratrol exerts anti-aging, anti-oxidation, anti-obesity, and other beneficial effects, and has been extensively used to treat cardiovascular diseases [26,27]. In mammalian studies, resveratrol has been reported to reduce the concentrations of both LDL-cholesterol and total cholesterol [28], increase bile acid excretion principally via elevating the expression of CYP7A1 [29], and promote the degradation of the apical sodium-dependent bile acid transporter [30]. These benefits were mainly fulfilled by targeting two nuclear receptors namely the FXRα and the liver X receptor α (LXRα), both of which precisely regulate glucose and lipid metabolism [24]. Recently, resveratrol has been demonstrated to increase the hepatic content of omega-3 fatty acids in fish-fed diets low in fish oil by elevating the expression of ∆6-desaturase [31,32]. In addition, it could relieve hypercholesterolemia in fish offered high-lipid or high-carbohydrate diets, therefore alleviating metabolic disorders [3,33,34]. These beneficial effects have been partly attributed to the antioxidant function of resveratrol [35]. Despite this, the molecular mechanisms underlying these physiological processes are still poorly illustrated. Whether it was correlated with an improved cholesterol homeostasis and bile acid metabolism is still unknown.

As an herbivorous freshwater carp, blunt snout bream (*Megalobrama amblycephala*) has a worldwide distribution and is cultivated widely in China [36]. To reduce the farming cost, diets formulated for this species often incorporate a substantial amount of carbohydrates. This inevitably results in several metabolic disorders especially fatty liver disease, thereby causing great economic loss [3]. Therefore, it is of great significance to illustrate the molecular mechanisms underlying the hepatobiliary syndrome, and meanwhile develop corresponding nutritional interventions to treat it, both of which unfortunately are still barely available. Thus, this study aimed to characterize the effects of feeding a high-carbohydrate diet on the redox response and the cholesterol and bile acid metabolism of *M. amblycephala*. Meanwhile, the beneficial effects of resveratrol supplementation were evaluated with the potential mechanisms unveiled. The results obtained here could facilitate the development of effective nutritional interventions to alleviate the glycolipid metabolism disorders in fish fed a high-energy low-protein feed. It might also reduce the cholesterol accumulation in fish products, thereby benefiting human health by avoiding excessive dietary cholesterol intake.

## 2. Materials and Methods

### 2.1. Ethics Statement

Fish were treated following the guidelines of the Care and Use of Laboratory Animals in China. The Animal Care and Use Committee at Nanjing Agricultural University (permit number: SYXK (Su) 2011-0036) has approved the present study.

### 2.2. Diets, Fish, and Sampling

Four isolipidic and isonitrogenous diets were formulated in the laboratory (Table 1), including a control diet (32% nitrogen-free extract, control), a high-carbohydrate diet (45% nitrogen-free extract, HC), and the HC diet supplemented with different doses of resveratrol (0.04%, HCR1; 0.08%, HCR2). The control and the HC diets were designated according to our previous study [36]. Resveratrol (98%) was purchased in Hubei Jusheng Technology Co., Ltd., (Hubei, China). The dosages were designated based on previous literature [3,33]. The ingredients were processed into diets through grinding, mixing, and granulating. The diets were then stored at −20 °C after drying.

The methods for determining the proximate composition of diets are shown below: the diets were dried at 105 °C until reaching a constant weight to determine the moisture content; the Kjeldahl method was used to determine the crude protein content (nitrogen × 6.25); the Soxtec SystemHT (Soxtec SystemHT6, Tecator, Höganäs, Sweden) was used to determine the crude lipid content by solvent extraction; the diets were burned in a muffle furnace for 5 h at 550 °C to determine the ash content; an adiabatic bomb calorimeter (PARR 1281, USA) was used to determine the gross energy content; an automatic analyzer (ANKOM A2000i, New York, United States) was used to determine the crude fiber content.

*M. amblycephala* juveniles were purchased in the National Fish Hatchery Station in Ezhou (Hubei, China). A total of 320 fish (35.0 ± 0.15 g) were reared in 16 floating cages in an outdoor pond after acclimation at a density of 20 individuals/cage. Then, the four experimental diets were artificially fed to fish three times daily (7:10, 12:10, and 16:10) until satiation for 12 weeks. After starvation for 24 h, all fish were weighed. Then four fish were selected randomly within each cage and were anesthetized by tricaine methanesulfonate (Sigma, Saint Louis, MO, USA) at 100 mg/L. Blood was rapidly sampled from the caudal vein, and was centrifuged at 3500 rpm at 4 °C for 15 min. Then the plasma was stored in a −80 °C refrigerator for biochemical analysis. Then, the hindgut and liver were both quickly removed and frozen rapidly in liquid nitrogen, and were kept at −80 °C for further use.

### 2.3. Measurements of Growth Performance and Tissue Metabolites Concentrations

The growth performance parameters were calculated as described by Shi et al. (2018) [3].

An ice-cold buffer (1 mM dithiothreitol, 1 mM EDTA, and 10 mM HEPES, pH 7.4) was used to homogenize both the liver and the hindgut. The concentrations of both triglyceride (TG) and total cholesterol (TC) in the tissue homogenate and plasma were determined following the procedures described by Tian et al. (2021) [37]. The concentrations of low-density lipoproteins cholesterol (LDL-C) and high-density lipoproteins cholesterol (HDL-C) in the plasma were both measured as described by Guo et al. (2019) [38]. The total bile acid (TBA) contents in the tissue homogenate and plasma were determined as described by Xu et al. (2020) [39]. Plasma glucose levels were determined as described by Asadi et al. (2009) [40]. Hepatic activities of catalase (CAT), glutathione peroxidase (GPX), and total superoxide dismutase (T-SOD) as well as the contents of malondialdehyde (MDA) and reduced glutathione (GSH) were all measured as described by Yang et al. (2019) [41]. The liver samples stored at −80 °C were homogenized to obtain the microsomes. Then the activity of CYP7A1 was detected by an ELISA kit for fish (MM-91713O2, Meimian, Yancheng, China).

### 2.4. Real-Time PCR (RT-PCR) and Western Blot

Total RNA was obtained from the liver and the hindgut by using the Trizol reagent (Invitrogen, CA, USA). The integrity of total RNA was determined by 1.0% agarose gel electrophoresis. The purity and quantity of the total RNA were checked using the spectrophotometric analysis at 260/280 nm. The RT-PCR kit (Takara, Dalian, China) was used for the cDNA synthesis following the instructions. Then, the levels of mRNA were determined by real-time PCR with the SYBR Green II Fluorescence Kit (Takara, Dalian, China). An ABI 7500 RT-q PCR system (Applied Biosystems, CA, USA) was used to complete the RT-PCR. The sequences and sources of the primers were both shown in Table 2. The elongation factor 1α (*ef1α*) was adopted to normalize the expressions of target genes following the 2^−ΔΔCt^ method [42,43].

Tissue lysate was prepared by mixing a radio-immunoprecipitation assay buffer (Thermo Fisher Scientific, Waltham, MA, USA) and the 100 mM phenylmethanesulfonyl fluoride at a proportion of 100:1. A proper amount of frozen tissue was then lysed with 1 mL of the tissue lysate, and was centrifuged at 10,000 rpm for 15 min. The total protein of the liver was extracted with its content analyzed by a bicinchoninic acid protein assay kit (Beyotime, Shanghai, China). The tissue lysate and SDS page (Beyotime, Shanghai, China) were both used to adjust the protein samples into equal concentrations. Subsequently, the samples were mixed, centrifuged, and heated at 99 °C for 8 min. Sodium dodecyl sulfate-polyacrylamide gelelectrophores were used to separate the protein, which was later transferred to polyvinylidene difluoridemembranes. The membranes were cut and sealed for 2 h, and were incubated overnight with primary antibodies, including the anti-Tubulin (55 kDa, 1:1000, #10094-1-AP, Proteintech, Wuhan, China), anti-LDLR (150–160 kDa, 1:1000, #10785-1-AP, Proteintech, Wuhan, China), anti-LXRα (45–50 kDa, 1:1000, #14351-1-AP, Proteintech, Wuhan, China), anti-SREBP2 (120–130 kDa, 1:1000, #28212-1-AP, Proteintech, Wuhan, China), anti-FXRα (56 kDa, 1:1000, #25055-1-AP, Proteintech, Wuhan, China), anti-HMGCR (95 kDa, 1:1000, #DF6518, Affinity, Changzhou, China) and anti-CYP7A1 (58 kDa, 1:1000, #DF2612, Affinity, Changzhou, China). The membranes were later incubated with the anti-rabbit (1:5000, #SA00001-2, Proteintech, Wuhan, China) secondary antibodies for 2 h on the next day. An enhanced chemiluminescent substrate (BL520B, Biosharp, Beijing, China) and aluminescent image analyzer (LAS-3000, Fujifilm, Tokyo, Japan) were used to detect and visualize the immune complexes, respectively. Then β-Tubulin was used to normalize the protein expressions. The ImageJ 1.44p software (U.S. National Institutes of Health, Bethesda, MD, USA) was adopted to quantify the intensity of each lane.

### 2.5. Statistical Analysis

Data were tested for normality and homoscedasticity by the SPSS program version 25.0 (SPSS Inc., Michigan Avenue, Chicago, IL, USA). The difference between the control group and the HC group was analyzed by the Student’s t-test. Meanwhile, differences among the HC, HCR1, and HCR2 groups were determined by one-way ANOVA using Tukey’s multiple-range test. The types (namely linear or quadratic) of significance were then determined by using the orthogonal polynomial contrasts. All data were presented as means ± S.E.M. (standard error of the mean) and were considered statistically significant at *p* < 0.05.

## 3. Results

### 3.1. Feed Utilization and Growth Performance

As shown in Table 3, dietary treatments exerted little influence (*p* > 0.05) on the final weight, WGR (weight gain rate), SGR (specific growth rate), feed intake, FCR (feed conversion ratio), and PER (protein efficiency ratio). However, the HSI (hepatosomatic index), VSI (viscera index) and IPF (intraperitoneal fat ratio) of the HC group were all remarkably (*p* < 0.05) higher than those of the control group. Meanwhile, the HSI decreased both linearly and quadratically (*p* < 0.05) in response to increasing dietary resveratrol levels.

### 3.2. Hepatic Antioxidant Capability

**Figure 1:**
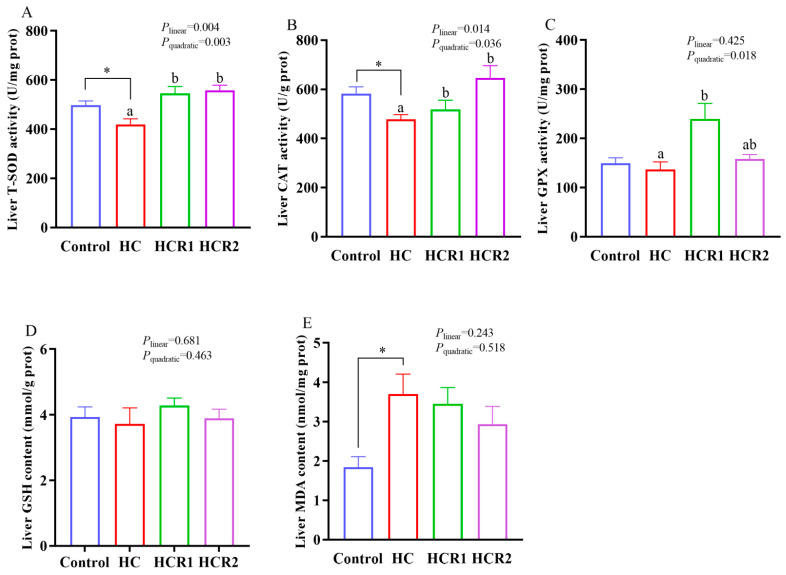
Liver T-SOD (**A**), CAT (**B**), and GPX (**C**) activities as well as GSH (**D**) and MDA (**E**) contents. Each data point represents the mean of four replicates (4 individuals per replicate). * Means *p* < 0.05, compared between the control and the HC group. ^ab^ Compared among the HC, HCR1, and HCR2 group. Bars assigned with different superscripts are significantly different (*p* < 0.05). Control, diet with 32% nitrogen-free extract; HC, diet with 45% nitrogen-free extract; HCR1, diet with 45% nitrogen-free extract and 0.04% resveratrol; HCR2, diet with 45% nitrogen-free extract and 0.08% resveratrol. MDA, malondialdehyde; CAT, catalase; GSH, L-glutathione reduced; GPX, glutathione peroxidase; T-SOD, total superoxide dismutase.

As can be seen from Figure 1, dietary treatments had no significant effect (*p* > 0.05) on the GSH content in the liver. However, the MDA content of the HC group was remarkably (*p* < 0.05) higher than that of the control group, while an opposite result was found in the activities of CAT and T-SOD. In addition, the activities of CAT and T-SOD increased both linearly and quadratically (*p* < 0.05), while GPX activity increased quadratically (*p* < 0.05) with increasing dietary resveratrol levels.

### 3.3. Tissue and Plasma Metabolites Concentrations

**Figure 2:**
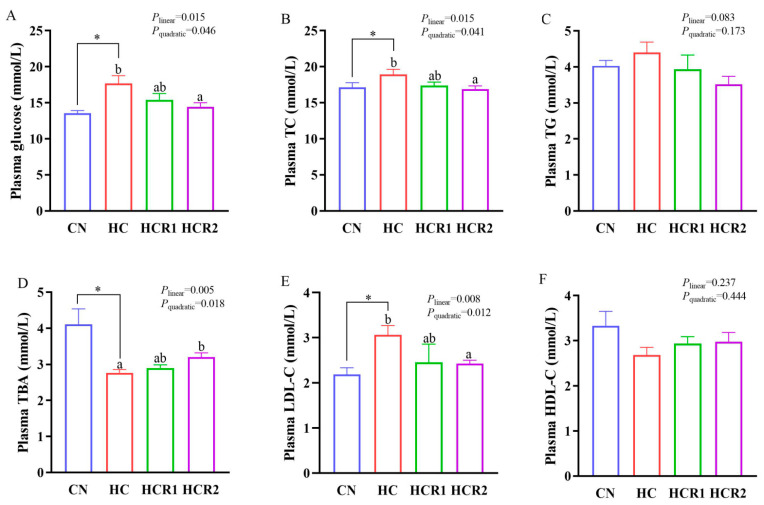
Plasma concentrations of glucose (**A**), TC (**B**), TG (**C**), TBA (**D**), LDL-C (**E**), and HDL-C (**F**). Each data point represents the mean of four replicates (4 individuals per replicate). * Means *p* < 0.05, compared between the control and the HC group. ^ab^ Compared among the HC, HCR1, and HCR2 groups. Bars assigned with different superscripts are significantly different (*p* < 0.05). Control, diet with 32% nitrogen-free extract; HC, diet with 45% nitrogen-free extract; HCR1, diet with 45% nitrogen-free extract and 0.04% resveratrol; HCR2, diet with 45% nitrogen-free extract and 0.08% resveratrol. TC, total cholesterol; TG, total triglycerides; TBA, total bile acid; HDL-C, high-density lipoproteins cholesterol; LDL-C, low-density lipoproteins cholesterol.

**Figure 3:**
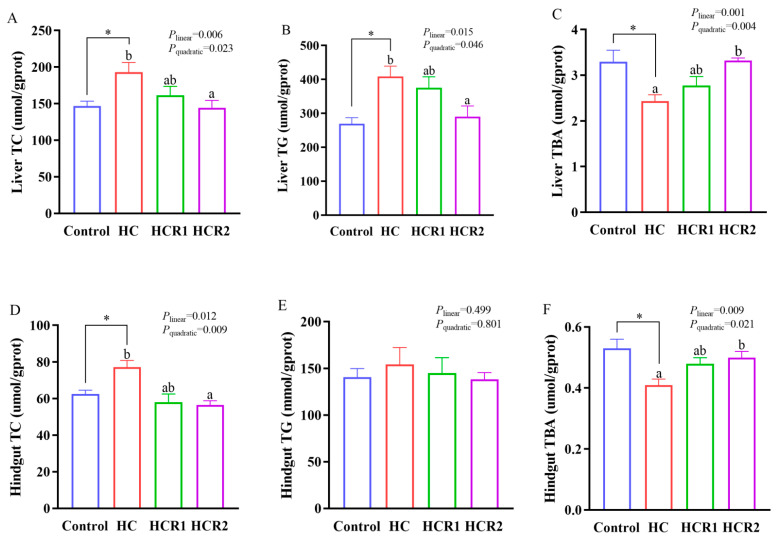
Liver TC (**A**), TG (**B**), and TBA (**C**) contents as well as hindgut TC (**D**), TG (**E**), and TBA (**F**) contents. Each data point represents the mean of four replicates (4 individuals per replicate). * Means *p* < 0.05, compared between the control and the HC group. ^ab^ Compared among the HC, HCR1, and HCR2 group. Bars assigned with different superscripts are significantly different (*p* < 0.05). Control, diet with 32% nitrogen-free extract; HC, diet with 45% nitrogen-free extract; HCR1, diet with 45% nitrogen-free extract and 0.04% resveratrol; HCR2, diet with 45% nitrogen-free extract and 0.08% resveratrol. TC, total cholesterol; TG, total triglycerides; TBA, total bile acid.

As presented in both Figure 2 and Figure 3, dietary treatments had no significant effect (*p* > 0.05) on the concentrations of TG and HDL-C in the plasma. However, the concentration of TG in the HC group was remarkably higher (*p* < 0.05) than that of the control group in the liver. A similar result was also found in the concentration of TC in the plasma, liver, and hindgut. In addition, tissue TC content and liver TG content decreased both linearly and quadratically (*p* < 0.05) with increasing dietary resveratrol levels. Plasma glucose and LDL-C concentrations also showed a similar result (*p* < 0.05), while the opposite was true for the TBA concentration (*p* < 0.05).

### 3.4. Transcriptions of the Genes Involved in Cholesterol and Bile Acid Metabolism and the Antioxidant Defense

**Figure 4:**
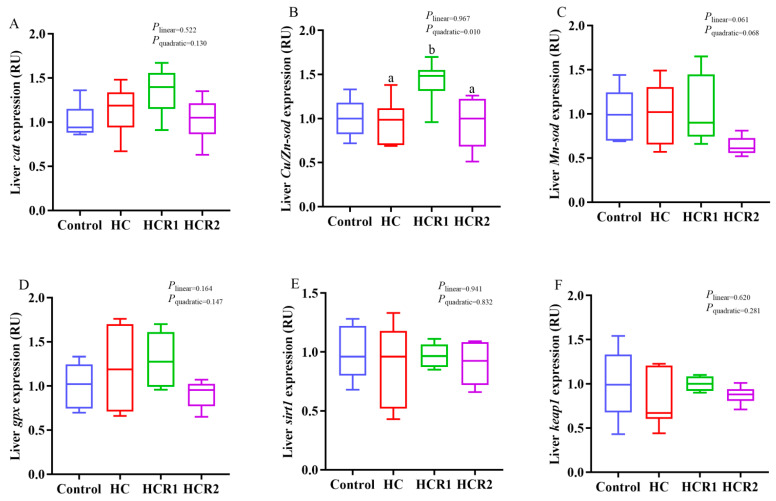
Hepatic transcriptions of the genes involved in the antioxidant defense. The mRNA levels of catalase (*cat*) (**A**), copper/zinc superoxide dismutase (*Cu/Zn-sod*) (**B**), manganese superoxide dismutase (*Mn-sod*) (**C**), glutathione peroxidase (*gpx*) (**D**), sirtuin 1 (*sirt1*) (**E**), kelch-like ECH associating protein 1 (*keap1*) (**F**) were evaluated using real-time RT-PCR. Data are referred to the values (relative units, RU) obtained in fish fed the control diet. Each data point represents the mean of four replicates (4 individuals per replicate). ^ab^ Compared among the HC, HCR1, and HCR2 groups. Bars assigned with different superscripts are significantly different (*p* < 0.05). The first and third quartiles are represented by the top and bottom of the box, while the minimum and maximum values are represented by the whiskers. Control, diet with 32% nitrogen-free extract; HC, diet with 45% nitrogen-free extract; HCR1, diet with 45% nitrogen-free extract and 0.04% resveratrol; HCR2, diet with 45% nitrogen-free extract and 0.08% resveratrol.

**Figure 5:**
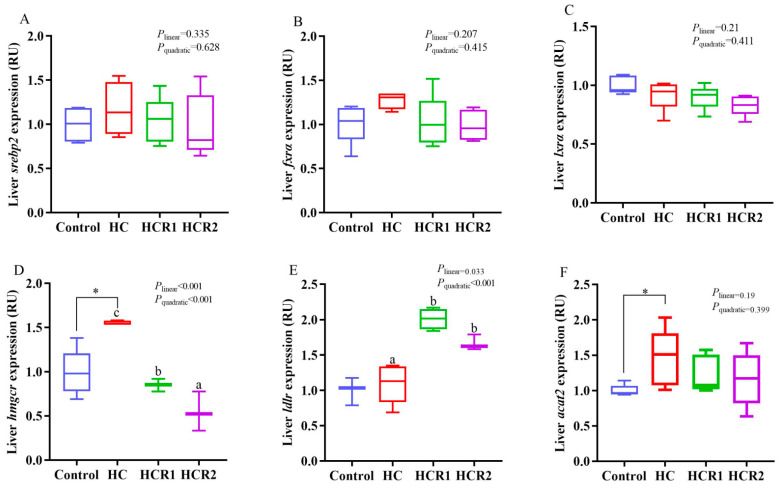
Hepatic transcriptions of the genes involved in cholesterol metabolism. The mRNA levels of sterol regulatory element-binding protein 2 (*srebp2*) (**A**), farnesoid X receptor α (*fxrα*) (**B**), liver X receptor α (*lxrα*) (**C**), 3-hydroxy-3-methyl-glutaryl coenzyme A reductase (*hmgcr*) (**D**), low-density lipoproteins receptor (*ldlr*) (**E**), acyl coenzyme A: cholesterol acyltransferase 2 (*acat2*) (**F**) were evaluated using real-time RT-PCR. Data are referred to the values (relative units, RU) obtained in fish fed the control diet. Each data point represents the mean of four replicates (4 individuals per replicate). * Means *p* < 0.05, compared between the control and the HC group. ^abc^ Compared among the HC, HCR1, and HCR2 groups. Bars assigned with different superscripts are significantly different (*p* < 0.05). The first and third quartiles are represented by the top and bottom of the box, while the minimum and maximum values are represented by the whiskers. Control, diet with 32% nitrogen-free extract; HC, diet with 45% nitrogen-free extract; HCR1, diet with 45% nitrogen-free extract and 0.04% resveratrol; HCR2, diet with 45% nitrogen-free extract and 0.08% resveratrol.

**Figure 6:**
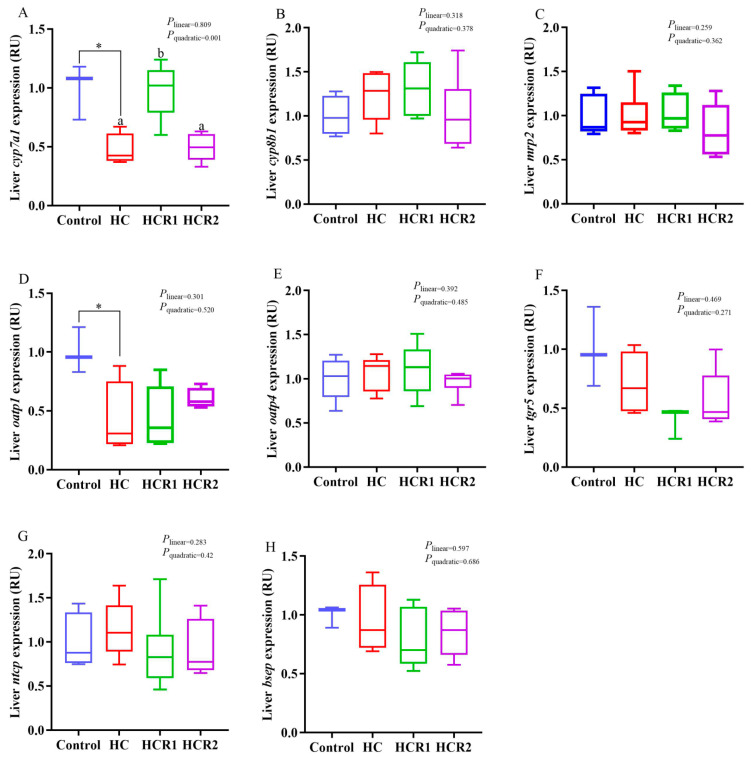
Hepatic transcriptions of the genes involved in bile acid metabolism. The mRNA levels of cholesterol 7α-hydroxylase (*cyp7a1*) (**A**), sterol 12α-hydroxylase (*cyp8b1*) (**B**), multidrug resistance associated protein 2 (*mrp2*) (**C**), organic anion-transporting polypeptide 1 (*oatp1*) (**D**), organic anion-transporting polypeptide 4 (*oatp4*) (**E**), takeda G-protein-coupled BA receptor (*tgr5*) (**F**), Na^+^-taurocholate cotransporter (*ntcp*) (**G**), bile salt export pump (*bsep*) (**H**) were evaluated using real-time RT-PCR. Data are referred to the values (relative units, RU) obtained in fish fed the control diet. Each data point represents the mean of four replicates (4 individuals per replicate). * Means *p* < 0.05, compared between the control and the HC group. ^ab^ Compared among the HC, HCR1, and HCR2 groups. Bars assigned with different superscripts are significantly different (*p* < 0.05). The first and third quartiles are represented by the top and bottom of the box, while the minimum and maximum values are represented by the whiskers. Control, diet with 32% nitrogen-free extract; HC, diet with 45% nitrogen-free extract; HCR1, diet with 45% nitrogen-free extract and 0.04% resveratrol; HCR2, diet with 45% nitrogen-free extract and 0.08% resveratrol.

As presented in Figure 4, Figure 5 and Figure 6, dietary treatments had no significant effect (*p* > 0.05) on the transcriptions of *lxrα*, *fxrα*, *srebp2*, *oatp4*, *bsep*, *mrp2*, *cyp8b1*, *tgr5*, *ntcp cat*, *Mn-sod*, *gpx*, *sirt1 and keap1* in the liver. However, the transcriptions of *hmgcr* and *acat2* in the HC group both increased remarkably (*p* < 0.05) compared with the control group, while that of *hmgcr* decreased both linearly and quadratically (*p* < 0.05) with increasing dietary resveratrol levels. Resveratrol treatment also up-regulated the transcription of *ldlr* in the HC group both linearly and quadratically (*p* < 0.05). The transcriptions of *cyp7a1* and *oatp1* in the control group both increased remarkably (*p* < 0.05) compared with the HC group, while resveratrol supplementation quadratically (*p* < 0.05) up-regulated the transcriptions of both *cyp7a1* and *Cu/Zn-sod* in the HC group.

**Figure 7:**
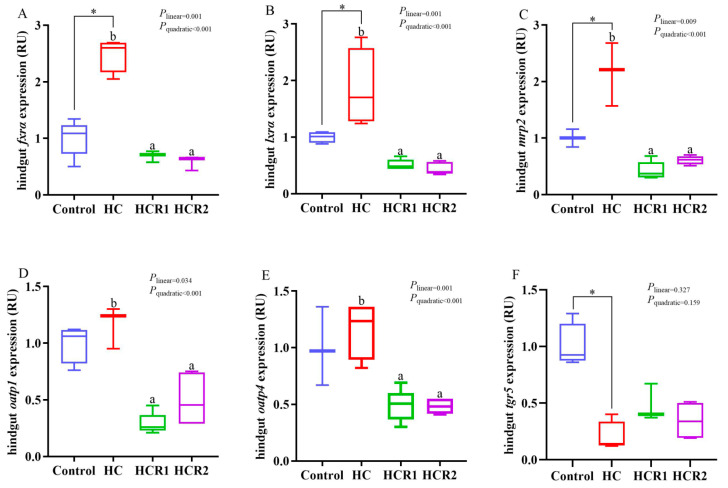
Relative transcriptions of the genes involved in cholesterol and bile acid metabolism in the hindgut. The mRNA levels of farnesoid X receptor α (*fxrα*) (**A**), liver X receptor α (*lxrα*) (**B**), multidrug resistance associated protein 2 (*mrp2*) (**C**), organic anion-transporting polypeptide 1 (*oatp1*) (**D**), organic anion-transporting polypeptide 4 (*oatp4*) (**E**), takeda G-protein-coupled BA receptor (*tgr5*) (**F**) were evaluated using real-time RT-PCR. Data are referred to the values (relative units, RU) obtained in fish fed the control diet. Each data point represents the mean of four replicates (4 individuals per replicate). * Means *p* < 0.05, compared between the control and the HC group. ^ab^ Compared among the HC, HCR1, and HCR2 groups. Bars assigned with different superscripts are significantly different (*p* < 0.05). The first and third quartiles are represented by the top and bottom of the box, while the minimum and maximum values are represented by the whiskers. Control, diet with 32% nitrogen-free extract; HC, diet with 45% nitrogen-free extract; HCR1, diet with 45% nitrogen-free extract and 0.04% resveratrol; HCR2, diet with 45% nitrogen-free extract and 0.08% resveratrol.

As presented in Figure 7, the transcriptions of *fxrα*, *lxrα*, and *mrp2* in the HC group were all remarkably (*p* < 0.05) higher than those in the control group in the hindgut. However, they all decreased both linearly and quadratically (*p* < 0.05) with increasing dietary resveratrol levels. The transcription of *tgr5* in the control group was remarkably (*p* < 0.05) higher than that in the HC group. In addition, the transcriptions of *oatp4* and *oatp1* in the HC group both exerted no statistical difference (*p* > 0.05) compared with the control group. However, they decreased both linearly and quadratically (*p* < 0.05) with increasing dietary supplementation levels.

### 3.5. Hepatic Contents of the Proteins Involved in Cholesterol and Bile Acid Metabolism as Well as the Cyp7a1 Activity

**Figure 8:**
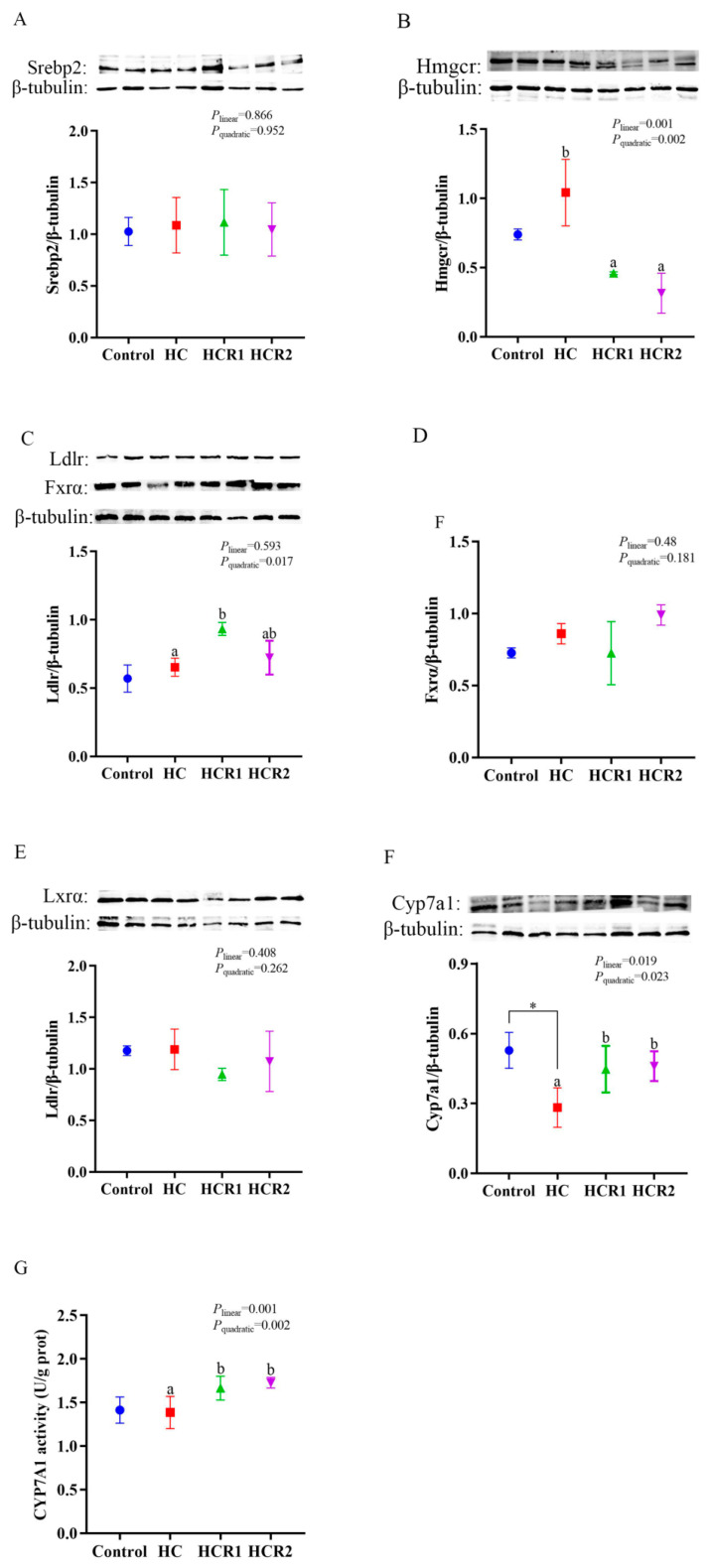
Protein levels of Srebp2 (**A**), Hmgcr (**B**), Ldlr (**C**), Fxrα (**D**), Lxrα (**E**), and Cyp7a1 (**F**) as well as the CYP7A1 (**G**) activity in the liver. Each data point represents the mean of four replicates (4 individuals per replicate). * Means *p* < 0.05, compared between the control and the HC group. ^ab^ Compared among the HC, HCR1, and HCR2 groups. Bars assigned with different superscripts are significantly different (*p* < 0.05). Control, diet with 32% nitrogen-free extract; HC, diet with 45% nitrogen-free extract; HCR1, diet with 45% nitrogen-free extract and 0.04% resveratrol; HCR2, diet with 45% nitrogen-free extract and 0.08% resveratrol. Srebp2, sterol regulatory element-binding protein 2; Hmgcr, 3-hydroxy-3-methyl-glutaryl coenzyme A reductase; Ldlr, low-density lipoproteins receptor; Fxrα, farnesoid X receptor α; Lxrα, liver X receptor; Cyp7a1, cholesterol 7α-hydroxylase.

As can be seen from Figure 8, dietary treatments had no significant effect (*p* > 0.05) on the protein contents of Srebp2, Lxrα, and Fxrα in the liver. The Hmgcr content decreased both linearly and quadratically (*p* < 0.05) with increasing dietary resveratrol levels. However, an opposite result was found in the content of Cyp7a1. The Ldlr content increased quadratically (*p* < 0.05) in response to increasing dietary resveratrol levels. Furthermore, the CYP7A1 activity in the HC group exerted no significant difference (*p* > 0.05) compared with the control group, but increased both linearly and quadratically (*p* < 0.05) with increasing dietary resveratrol levels.

## 4. Discussion

In the control diet, microcrystalline cellulose as a filler was used at 14%, which is within the safe range for *M. amblycephala* [36,44]. Actually, being an herbivorous carp, this species has a high tolerance for dietary cellulose compared with most omnivorous and carnivorous ones. In fact, no growth retardation was observed in this species fed a diet incorporating 21% cellulose [36]. In addition, dietary treatments exerted little influence on the growth performance of *M. amblycephala*. However, feeding the HC diet induced a remarkable increase in the HSI, VSI, and IPF compared with the control group, while resveratrol supplementation significantly decreased the HSI. This result is justifiable since excessive carbohydrate intake inevitably results in lipid accumulation in the liver of fish, while resveratrol could alleviate it by promoting lipolysis [34]. It is obvious that feeding the HC diet induced a remarkable increase in MDA content coupled with the decreased activities of both CAT and T-SOD in the liver. These results demonstrated that ong-term feeding of the HC diet decreased the antioxidant ability of *M. amblycephala*. Generally, the activities of antioxidant enzymes like SOD and CAT can directly reflect the antioxidant capability of fish, while the MDA content is positively correlated with oxidative damage [45]. In addition, dietary resveratrol supplementation remarkably increased the activities of CAT, GPX and T-SOD coupled with the up-regulated transcription of *Cu/Zn-sod*, demonstrating that resveratrol could enhance the antioxidant capacity of fish.

Resveratrol, an antioxidant and bioactive molecule, plays a key role in reliving hypercholesterolemia in mammals [46]. However, the relevant information is still absent in aquatic species. In this study, feeding the HC diet remarkably elevated tissue TC (in the plasma, liver, and hindgut) and TG (in the liver) concentrations in *M. amblycephala*. This also held true for plasma glucose and LDL-C concentrations. However, resveratrol supplementation remarkably decreased their concentrations. This might suggest that high dietary carbohydrate levels resulted in both hypercholesterolemia and hypertriglyceridemia in this species, while resveratrol administration could alleviate it. As mentioned earlier, the intake of an HC diet could eventually promote lipogenesis and lipoidosis in fish, thereby generating both hypercholesterolemia and hypertriglyceridemia [3,36]. However, resveratrol could reduce the circulating levels of TC and TG, thereby preventing liver steatosis in overfed rodents and zebrafish (*Danio rerio*) [34,47]. Indeed, in this study, high dietary carbohydrate levels remarkably elevated the hepatic expressions of both *hmgcr* and *acat2*, which are vital enzymes in cholesterol synthesis and esterification, respectively [11]. Meanwhile, resveratrol supplementation remarkably decreased the transcriptions of both *hmgcr* and *acat2* as well as the protein content of Hmgcr in the liver. In addition, resveratrol supplementation remarkably increased both the transcription and protein content of hepatic Ldlr compared with the HC group. This might indicate that resveratrol administration could promote cholesterol uptake by the liver, thereby enhancing cholesterol catabolism in fish. Generally, cholesterol is mainly taken from the circulation system by LDLR in the peripheral cells as a component of LDLs [11]. Supportively, a decreased HMGCR level coupled with an increased LDLR content was both observed in the liver of rat off-springs delivered by the female rats fed resveratrol during lactation [48]. Furthermore, in this study, the HC diet significantly increased the transcription of *lxrα* in the hindgut, while an opposite result was noticed after resveratrol supplementation. This also held true for the transcription and protein content of Lxrα in the liver, although no significant difference was noticed. This again reinforced the fact that high dietary carbohydrate levels induced lipogenesis in *M. amblycephala*, while resveratrol administration could alleviate it. Generally, activated LXR could enhance lipogenesis through the induction of SREBPs [49]. Supportively, LXR agonists could increase lipogenesis, and induce fatty liver in rodents [50], while resveratrol supplementation could alleviate both syndromes [51]. Accordingly, it might be speculated that resveratrol could regulate the cholesterol metabolism in fish by targeting the intestinal LXRα. Together, it might be inferred that a long-period consumption of the HC diet could increase the cholesterol synthesis in *M. amblycephala*. However, resveratrol supplementation enhanced cholesterol catabolism by inhibiting cholesterol biosynthesis while promoting cholesterol uptake in the liver. This might be advanced to interpret the results obtained in the plasma and tissue metabolites in the present study.

In this study, the HC diet remarkably decreased the concentration of TBA in the plasma, liver, and hindgut of *M. amblycephala*, while the opposite was true by resveratrol treatment. Consistently, similar results were also noted in both the transcription and protein content of Cyp7a1 in the liver. Furthermore, resveratrol administration also significantly increased the activity of CYP7A1 in the liver compared with the HC group. These results indicated that long-term consumption of the HC diet could decrease the bile acid synthesis in *M. amblycephala*, whereas the supplementation of resveratrol could alleviate this syndrome. Generally, as the rate-limiting enzyme of bile acid synthesis, CYP7A1 could promote the conversion of cholesterol into bile acids in the liver, as is mediated by the classical neutral pathway [11]. Supportively, resveratrol intake remarkably elevated the CYP7A1 expression in HepG2 cells and increased the size of the bile acid pool in mice [29]. In addition, compared with the control group, the HC diet remarkably decreased the transcription of *oatp1* in the liver, while the transcription of *mrp2* in the hindgut showed an opposite result. However, in the hindgut, the supplementation of resveratrol decreased the transcriptions of *oatp1*, *oatp4,* and *mrp2*, all of which are bile acids transporters in the enterohepatic circulation [17]. This might suggest that long-term consumption of the HC diet could inhibit the bile acids reabsorption, and increase the biliary excretion in *M. amblycephala*, thereby resulting in the decreased TBA concentration. This actually might be a defense mechanism for fish to cope with the enhanced cholesterol synthesis caused by the intake of the HC diet. However, resveratrol supplementation could decrease the bile acid reabsorption, and increase the bile acid synthesis and biliary excretion, thereby leading to increased TBA concentration and reduced TC level. It is widely acknowledged that bile acids are transported by the OATPs into cells, then are secreted into both gallbladder and the small intestine with the assistance of MRP2 [17]. Supportively, previous studies have shown that polyphenols such as resveratrol can increase bile acid excretion via increasing the expression of CYP7A1 and reducing the expression of bile acid transporters in the intestine, thereby reducing the circulating levels of both TC and LDL-C [52,53]. Supportively, cholestyramine administration markedly reduced the transcription of *MRP2* in the intestine of rats, thereby maintaining cholesterol homeostasis [54].

Like LXR, FXR is also closely involved in bile acid metabolism. In this study, the HC diet significantly increased the transcription of *fxrα* in the hindgut after a 12-week feeding trial, while the opposite was true by resveratrol treatment. Similar results were also noticed in both the transcription and protein levels of Fxr in the liver, although no significant difference was noted. It was speculated that the HC diet might increase the content of certain bile acids, which have a strong affinity for the intestinal *fxrα*, thereby activating it. However, resveratrol treatment deactivated *fxrα* via modulating these bile acids. This was supported by the fact that bile acids are natural agonists of FXR, and different bile acids have distinct affinities for FXRα. Generally, chenodeoxycholic acid exerts the highest affinity for FXR followed by deoxycholic acid and cholic acid [17]. In addition, the inhibited transcription of *fxrα* by resveratrol treatment might also be ascribed to the activation of SIRT1. Indeed, it has been demonstrated that resveratrol treatment could activate SIRT1, which consequently reduced the acetylation level of FXR, thereby decreasing its activity [55]. However, this information is derived from a mouse. Further in-depth investigations into aquatic animals are needed to confirm this. Furthermore, it has been demonstrated that *cyp7a1*, *oatp1*, *oatp4*, and *mrp2* are all target genes of FXR, and are involved in virtually every part of the bile acid cycle [17]. Taken together, it might be speculated that the HC diet remarkably increased the transcription of *fxrα* in the hindgut, consequently reducing the transcriptions of *oatp1* and *cyp7a1* in the liver, and increased the transcription of *mrp2* in the hindgut, thereby reducing both the synthesis and flow of bile acids. However, resveratrol treatment decreased the transcription of *fxrα*, which might in turn increase the transcription of *cyp7a1*, and down-regulate the transcriptions of *oatp1*, *oatp4,* and *mrp2*, thereby increasing both the synthesis and excretion of bile acids. This might ultimately improve cholesterol homeostasis, and reduce the lipid accumulation in *M. amblycephala* offered the HC diet.

Furthermore, in the present study, the HC diet significantly decreased the transcription of *tgr5* in the hindgut, while the opposite was true after resveratrol supplementation. This might suggest that a long-time consumption of the HC diet caused dysfunctions in the glucose metabolism in *M. amblycephala*, while reservatrol administration alleviated it. Supportively, it has been reported that the activation of Tgr5 could increase energy expenditure and the release of glucagon-like peptide-1 in obese mice [56]. This could in turn enhance glucose tolerance and improve the functions of both the liver and the pancreas, as might ultimately lead to enhanced insulin sensitivity and improved glucose homeostasis [56].

## 5. Conclusions

In summary, high dietary carbohydrate levels decreased the antioxidant capability and bile acid synthesis of blunt snout bream but enhanced the cholesterol synthesis via up-regulating both *hmgcr* and *acat2*, and down-regulating T-SOD, CAT, and *cyp7a1*, thereby resulting in excessive cholesterol accumulation. Resveratrol treatment enhanced the antioxidant capability by up-regulating T-SOD, CAT, GPX, and *Cu/Zn-sod*, decreased cholesterol synthesis and uptake in the liver by down-regulating both *hmgcr* and *acat2*, and up-regulating *ldlr*. It also increased bile acid synthesis and biliary excretion by up-regulating *cyp7a1*, and down-regulating *mrp2*, *oatp1,* and *oatp4* in the hindgut, thereby decreasing cholesterol accumulation in fish.

## Figures and Tables

**Table 1:** Formulation and proximate composition of the experimental diets.

Ingredients (%)	Control	HC	HCR1	HCR2
Fish meal	5.10	5.10	5.10	5.10
Soybean meal	29.59	29.59	29.59	29.59
Rapeseed meal	15.31	15.31	15.31	15.31
Cottonseed meal	15.31	15.31	15.31	15.31
Fish oil	2.04	2.04	2.04	2.04
Soybean oil	2.04	2.04	2.04	2.04
Corn starch	13.27	27.55	27.55	27.55
Microcrystalline cellulose	14.28	0.00	0.00	0.00
Resveratrol	0.00	0.00	0.04	0.08
Calcium biphosphate	2.04	2.04	2.04	2.04
Premix ^1^	1.02	1.02	1.02	1.02
Proximate composition (% air-dry basis)				
Moisture	10.6	11.3	10.7	10.7
Crude protein	29.9	29.1	29.3	29.0
Crude lipid	5.3	5.2	5.4	5.5
Ash	6.6	6.5	6.4	6.6
Crude fiber	16.0	3.5	3.5	3.6
Nitrogen-free extract ^2^	31.6	44.4	44.7	44.6
Energy (MJ/kg)	18.5	18.0	17.8	18.4

Control, diet with 32% nitrogen-free extract; HC, diet with 45% nitrogen-free extract; HCR1, diet with 45% nitrogen-free extract and 0.04% resveratrol; HCR2, diet with 45% nitrogen-free extract and 0.08% resveratrol. ^1^ Premix supplied the following minerals and vitamins (per kg): CuSO_4_·5H_2_O, 2.0 g; FeSO_4_·7H_2_O, 25 g; ZnSO_4_·7H_2_O, 22 g; MnSO_4_·4H_2_O, 7.0 g; Na_2_SeO_3_, 0.04 g; KI, 0.026 g; CoCl_2_·6H_2_O, 0.1 g; Vitamin A, 900,000 IU; Vitamin D, 200,000 IU; Vitamin E, 4500 mg; Vitamin K_3_, 220 mg; Vitamin B_1_, 320 mg; Vitamin B_2_, 1090 mg; Vitamin B_5_, 2000 mg; Vitamin B_6_, 500 mg; Vitamin B_12_, 1.6 mg; Vitamin C, 5000 mg; Pantothenate, 1000 mg; Folic acid, 165 mg; Choline, 60,000 mg. ^2^ Calculated by difference (100 − moisture − crude protein − crude lipid − ash − crude fiber).

**Table 2:** Nucleotide sequences of the primers used to assay gene expressions by real-time PCR.

Target Genes	Forward (5′-3′)	Reverse (5′-3′)	Accession Numbers
*fxrα*	CACCAAGAAACTGCCCAACG	CTGTGTCACTGAACGTCCCA	MZ506835
*lxrα*	AACGTGCAGGATCACGAGTT	GCTTCATCAGCATACGGGGA	NC_063044.1
*cyp7a1*	ATATGATCAGGTGCCCTGCG	TCGTGCACAGCAAAGAAACG	XM_048164491.1
*cyp8b1*	AAACAGGACAGGGGGCAAAA	CCCTCGCGGATCTTGTACTC	NC_063044.1
*srebp2*	CTCAGCTTTCTGCCGGGTTA	TCACCGACCAATCACAGCTC	NC_063044.1
*hmgcr*	CGCAGAAAAATGCTCACCCC	CATGGGGACCTGAAACTGCT	XM_048189198.1
*acat2*	CCCGAAGAAGGTTCGCCTTA	TCTTTCAGGCCCGTCACATC	XM_048206846.1
*ldlr*	TCATTCCCCGCCTGAAGAAC	GGGTCGACCACTATAGCACG	XM_048158987.1
*cyp27a1*	AGGGTGACAGAGTCCCAACA	GCGGTCAGGTTTGAACTTTCG	NC_063044.1
*tgr5*	CCATCCTTTCCATTGCTGCG	CGCATTTCCAGTCTCCGTCT	NC_063044.1
*mrp2*	GTTCGATACCGGCCTGAGTT	CCAGTTCGACCAACAATGCC	NC_063044.1
*bsep*	GGCCGTGAATCTACTAAGGTCA	GGCTCCTGAGACACAATTCCA	NC_063044.1
*ntcp*	GATCCCAATACCGCAGGGAA	ATGCCCCTCCTGCTCTTAGT	NC_063044.1
*oatp1*	TCGGTCCAGTGTTTGGCTAC	TCCTGGGGTGATGCTAATGC	NC_063044.1
*oatp4*	GGGCAGCTACATGAAAAGCTC	ATCCAATCAGCGAGCTGGGAAT	NC_063044.1
*cat*	CAGTGCTCCTGATACCCAGC	TTCTGACACAGACGCTCTCG	XM_048158628.1
*Cu/Zn-sod*	AGTTGCCATGTGCACTTTTCT	AGGTGCTAGTCGAGTGTTAGG	KF479046.1
*Mn-sod*	AGCTGCACCACAGCAAGCAC	TCCTCCACCATTCGGTGACA	KF195932.1
*gpx*	GAACGCCCACCCTCTGTTTG	CGATGTCATTCCGGTTCACG	KF378713.1
*sirt1*	TCGGTTCATTCAGCAGCACA	ATGATGATCTGCCACAGCGT	MT518159.1
*keap1*	AATATCCGCCGGCTGTGTAG	TGAGTCCGAGGTGTTTCGTG	XM_048200093.1
*ef1α*	CTTCTCAGGCTGACTGTGC	CCGCTAGCATTACCCTCC	X77689.1

*fxrα*, farnesoid X receptor α; *lxrα*, liver X receptor; *cyp7a1*, cholesterol 7α-hydroxylase; *cyp8b1*, sterol 12α-hydroxylase; *srebp2*, sterol regulatory element-binding protein 2; *hmgcr*, 3-hydroxy-3-methylglutaryl-CoA reductase; *acat2*, acyl coenzyme A:cholesterolacyltransferases; *ldlr*, low-density lipoprotein receptor; *cyp27a1*, sterol 27-hydroxylase; *tgr5*, takeda G-protein-coupled BA receptor; *mrp2*, multidrug resistance associated protein 2; *bsep*, bile salt export pump; *ntcp*, Na^+^-taurocholate cotransporter; *oatp1*, organic anion-transporting polypeptide 1; *oatp4*, organic anion-transporting polypeptide 4; *cat*, catalase; *Cu/Zn-sod*, copper/zinc superoxide dismutase; *Mn-sod*, manganese superoxide dismutase; *gpx*: glutathione peroxidase; *sirt1*, sirtuin 1; *keap1*, kelch- like ECH associating protein 1; *ef1α*, elongation factor 1α.

**Table 3:** Growth performance and feed utilization of blunt snout bream fed different experimental diets.

Parameters	Control	HC	HCR1	HCR2	Polynomial Contrasts	
					Linear	Quadratic
**Initial weight (g)**	35.00 ± 0.15	34.97 ± 0.03	34.90 ± 0.17	35.03 ± 0.06	ns	ns
**Final weight (g)**	233.50 ± 28.50	217.17 ± 14.35	220.52 ± 20.52	204.40 ± 12.95	ns	ns
**WGR (%) ^1^**	566.81 ± 86.07	521.13 ± 41.42	531.89 ± 58.73	483.42 ± 36.80	ns	ns
**SGR (%/day) ^2^**	2.33 ± 0.16	2.25 ± 0.09	2.26 ± 0.12	2.17 ± 0.08	ns	ns
**Feed intake (g/fish) ^3^**	298.40 ± 39.40	267.89 ± 10.60	281.51 ± 26.12	267.35 ± 11.13	ns	ns
**FCR ^4^**	1.51 ± 0.02	1.48 ± 0.06	1.52 ± 0.05	1.59 ± 0.09	ns	ns
**PER ^5^**	2.22 ± 0.03	2.34 ± 0.10	2.25 ± 0.07	2.19 ± 0.13	ns	ns
**HSI (%) ^6^**	1.44 ± 0.07	1.79 ± 0.07 *^b^	1.65 ± 0.05 ^ab^	1.54 ± 0.05 ^a^	0.04	0.017
**VSI (%) ^7^**	9.58 ± 0.27	10.53 ± 0.31 *	10.22 ± 0.44	10.22 ± 0.27	ns	ns
**IPF (%) ^8^**	1.84 ± 0.15	2.68 ± 0.14 *	2.86 ± 0.31	2.36 ± 0.20	ns	ns

Control, diet with 32% nitrogen-free extract; HC, diet with 45% nitrogen-free extract; HCR1, diet with 45% nitrogen-free extract and 0.04% resveratrol; HCR2, diet with 45% nitrogen-free extract and 0.08% resveratrol.* Means *p* < 0.05, compared between the control and the HC group. ^ab^ Compared among the HC, HCR1, and HCR2 groups. Means in the same line with different superscripts are significantly different (*p* < 0.05). ns, not significant. ^1^ Weight gain rate (WGR) = (W_t_ − W_0_) × 100/W_0_, ^2^ Specific growth rate (SGR) = (LnW_t_ − LnW_0_) × 100/T, W_0_ and W_t_ mean the initial and final weights, respectively, T means the number of breeding days. ^3^ Feed intake = Total feed intake (g)/total fish number. ^4^ Feed conversion ratio (FCR) = Feed consumption (g)/fish weight gain (g). ^5^ Proteinefficiencyratio (PER) = Fish weight gain (g)/total protein fed (g). ^6^ Hepatosomatic index (HSI) = Liver weight (g) × 100/body weight (g). ^7^ Viscera index (VSI) = Viscera weight (g) × 100/body weight (g). ^8^ Intraperitoneal fat ratio (IPF) = (Intraperitoneal fat weight (g) × 100/body weight (g).

## Data Availability

Data are contained within the manuscript.
